# Correction: cytoNet: Spatiotemporal network analysis of cell communities

**DOI:** 10.1371/journal.pcbi.1010644

**Published:** 2022-11-08

**Authors:** PLOS Computational Biology

The contrast in Fig 1 was erroneously altered during publication. Please view the correct Fig 1 here. The publisher apologizes for the error.

**Fig 1:**
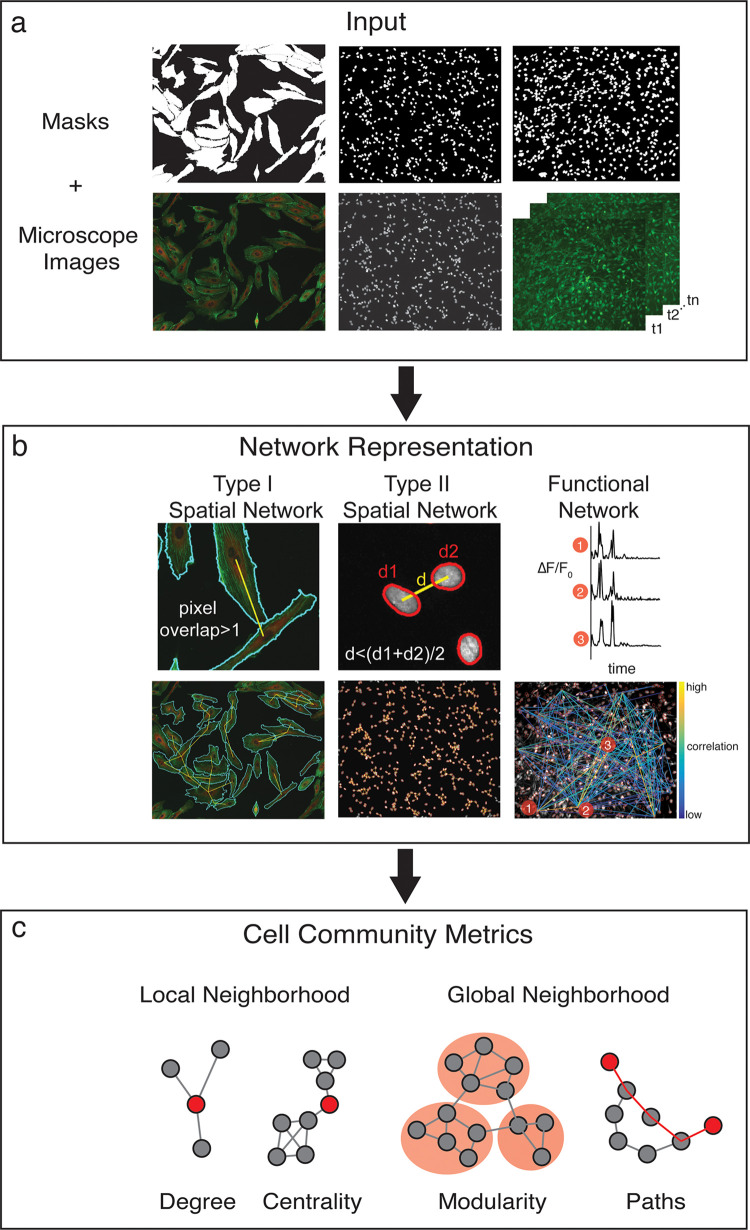
cytoNet workflow. (a) The cytoNet pipeline begins with masks and optionally microscope images, which can be static immunofluorescence images or calcium image sequences. (b) Spatial proximity is determined either by measuring shared pixels between cell pairs–type I networks, or by comparing the distance between cell centroids to a threshold distance–type II networks (right panel). Functional networks are estimated from correlations in calcium time series data. (c) Cell community descriptors provide information on local neighborhood characteristics of individual cells, like degree and centrality measures, and global neighborhood characteristics like modularity and path lengths.

## References

[1] Mahadevan AS, Long BL, Hu CW, Ryan DT, Grandel NE, et al. (2022) cytoNet: Spatiotemporal network analysis of cell communities. PLOS Computational Biology 18(6): e1009846. 10.1371/journal.pcbi.1009846 35696439 PMC9191702

