# Correction: cytoNet: Spatiotemporal network analysis of cell communities

**DOI:** 10.1371/journal.pcbi.1013176

**Published:** 2025-06-10

**Authors:** Arun S. Mahadevan, Byron L. Long, Chenyue W. Hu, David T. Ryan, Nicolas E. Grandel, George L. Britton, Marisol Bustos, Maria A. Gonzalez Porras, Katerina Stojkova, Andrew Ligeralde, Hyeonwi Son, John Shannonhouse, Jacob T. Robinson, Aryeh Warmflash, Eric M. Brey, Yu Shin Kim, Amina A. Qutub

The following information is missing from the Funding statement: NIH 5T32DE014318 grant to JS.
