# Supplementary figures and images for: cytoNet: Spatiotemporal network analysis of cell communities
Source: PLoS Comput Biol. 2022 Jun 13;18(6):e1009846. doi: 10.1371/journal.pcbi.1009846 (PMC9191702; doi:10.1371/journal.pcbi.1009846)

a

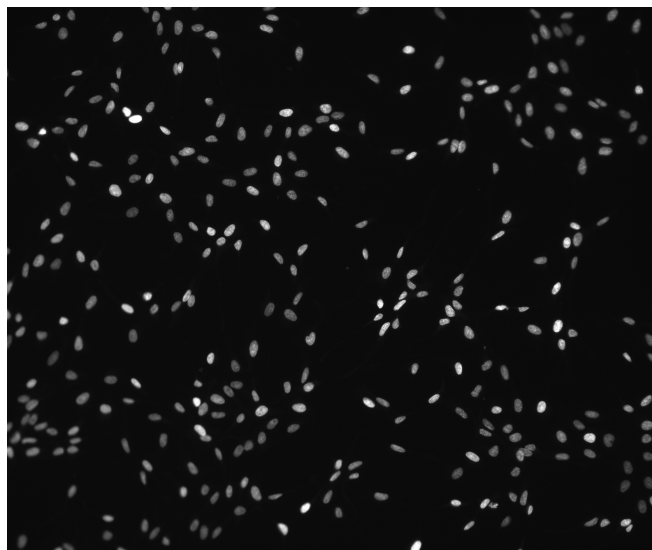

b

Original Graph

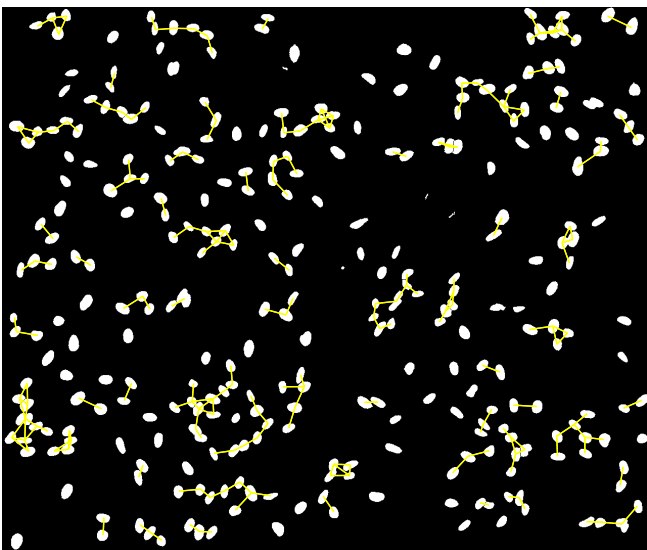

c

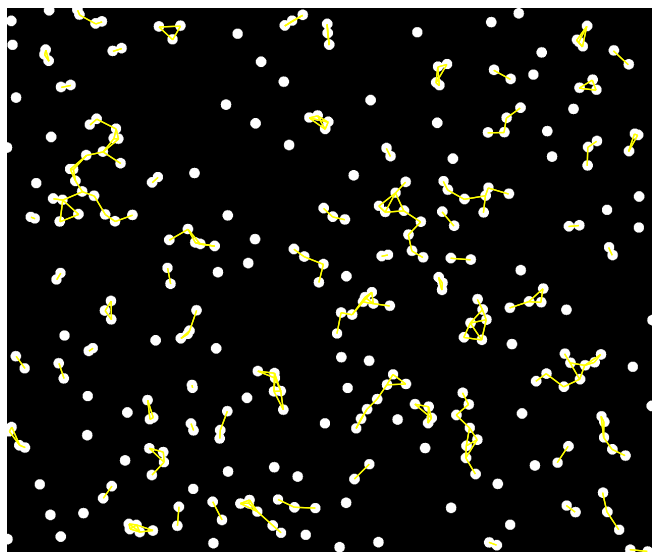

Spatial Random Graph 1

d

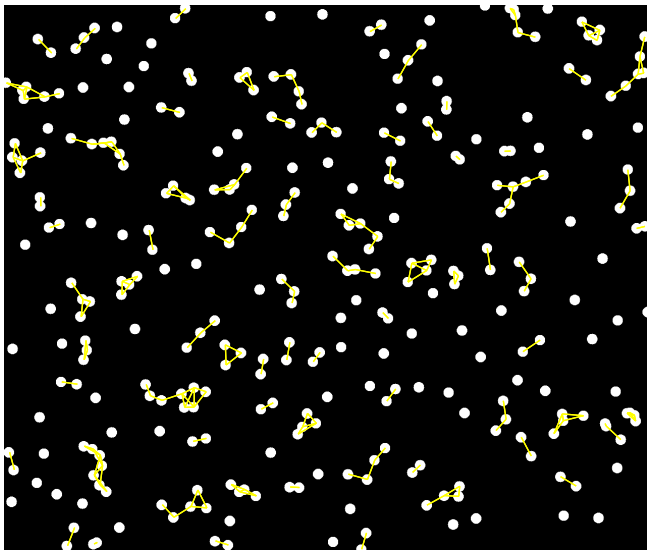

Spatial Random Graph 2

Supplement: S1 Fig — (a) Original grayscale image. (b) Binary mask with overlaid spatial graph. (c) Random graph created by placing spheres of diameter equal to the average equivalent diameter of all ROIs in (b) at random locations and reapplying connection rules. (d) Second iteration of random graph creation. (PDF) [file pcbi.1009846.s001.pdf]

a

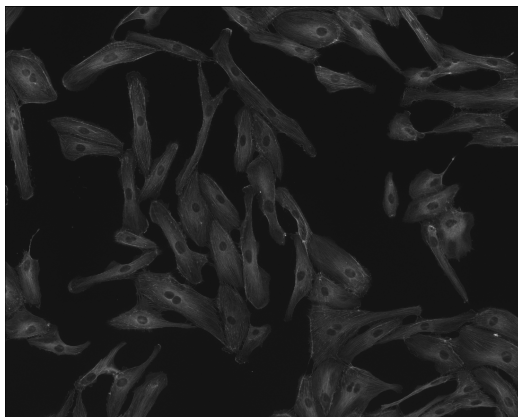

b

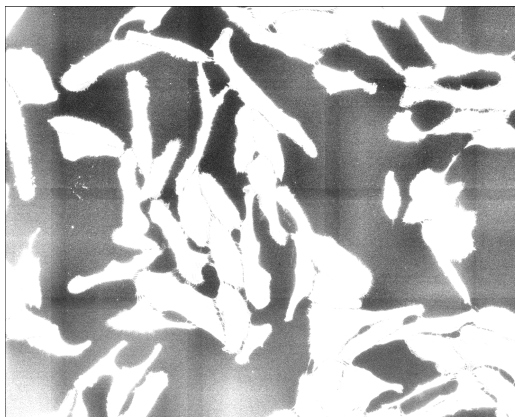

c

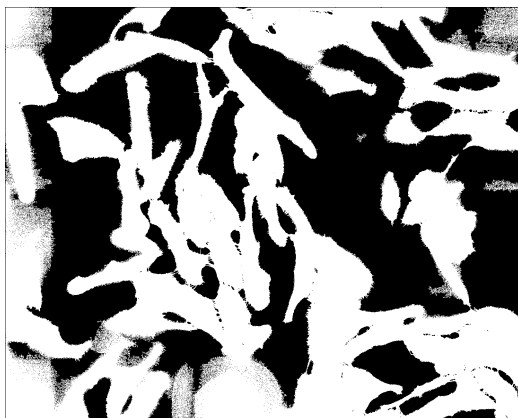

d

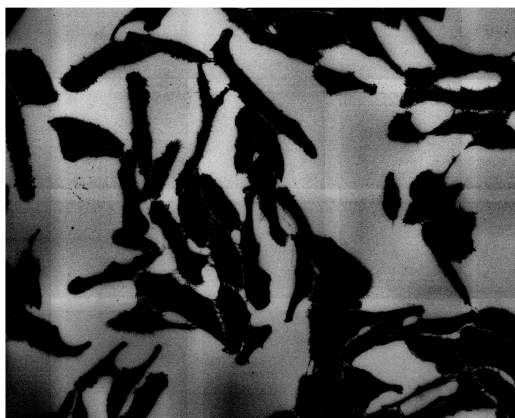

e

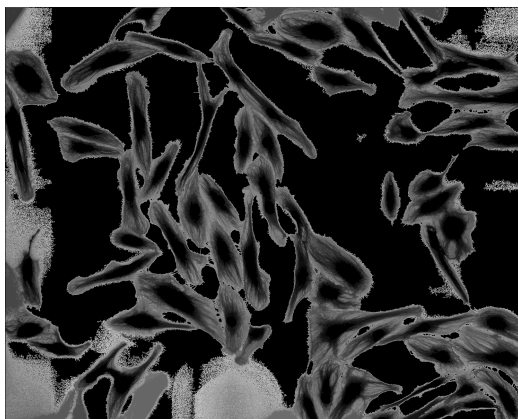

f

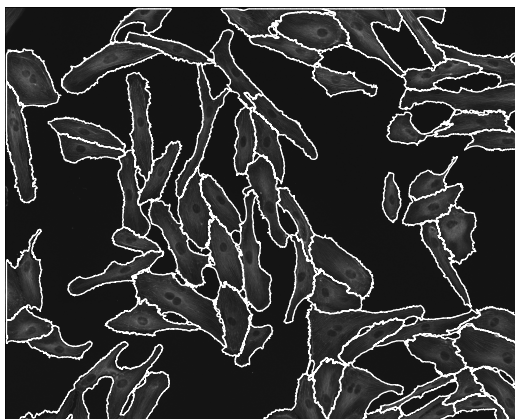

Supplement: S2 Fig — (a) Original grayscale image. (b) Image after adaptive histogram equalization and Gaussian filtering. (c) Binary image obtained using Otsu’s threshold, with small objects removed. (d) Complement of filtered image in (b). (e) Watershed basins obtained through imposing minimum of images in (d) and the marker image (obtained by combining the binary image in (c) and the image obtained through binarization of microtubules and nuclei). (f) Final cell borders. (PDF) [file pcbi.1009846.s002.pdf]

a

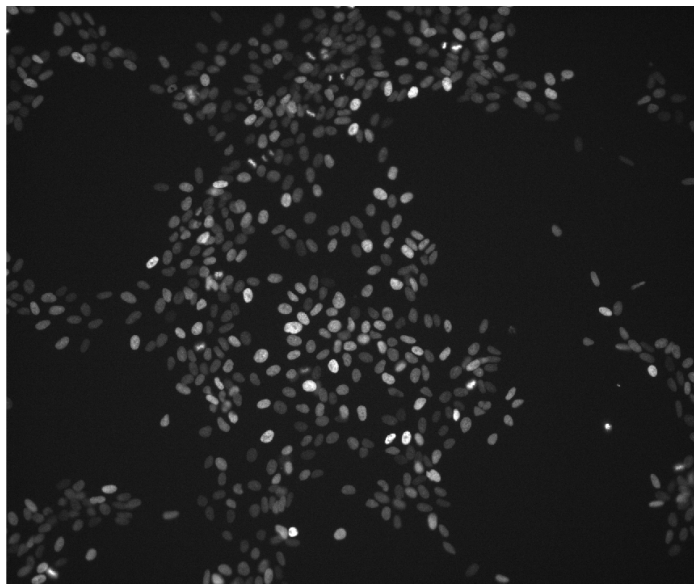

b

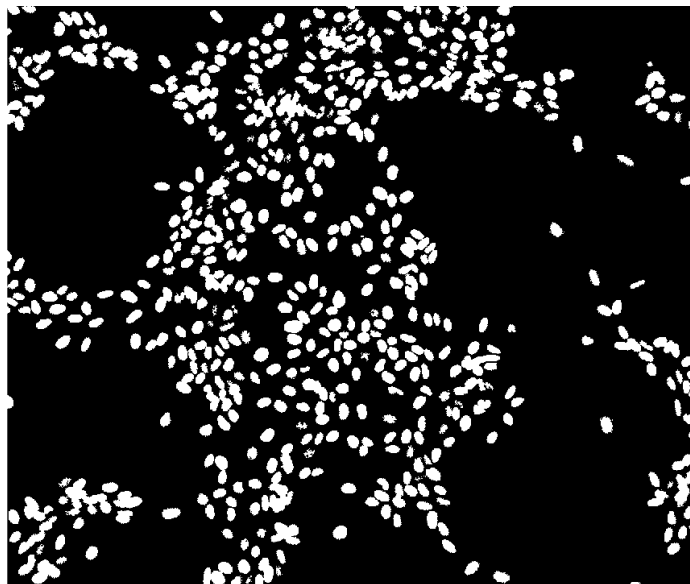

c

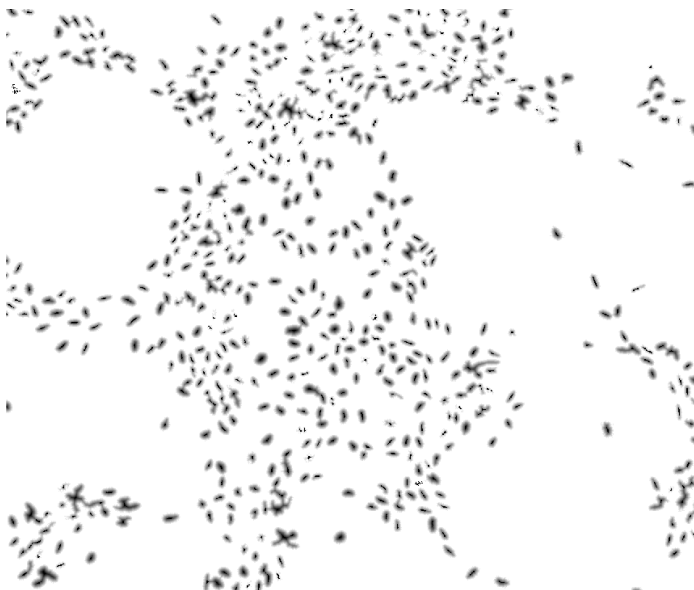

d

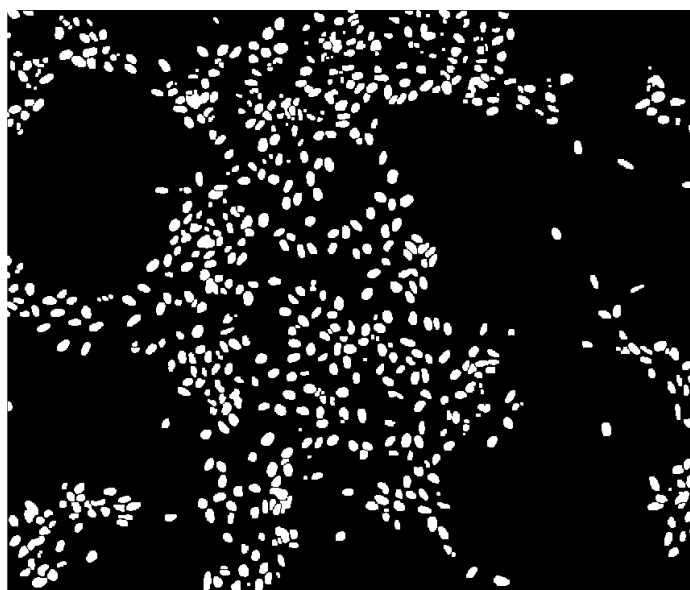

Supplement: S3 Fig — (a) Fluorescence image from H2B-Cerulean channel marking all nuclei. (b) Binary mask obtained through adaptive thresholding. (c) Image obtained through imposing minimum of distance transform of binary image in (b) and local minima. This image serves as seeds for the watershed algorithm. (d) Final mask obtained after watershed transform. (PDF) [file pcbi.1009846.s003.pdf]

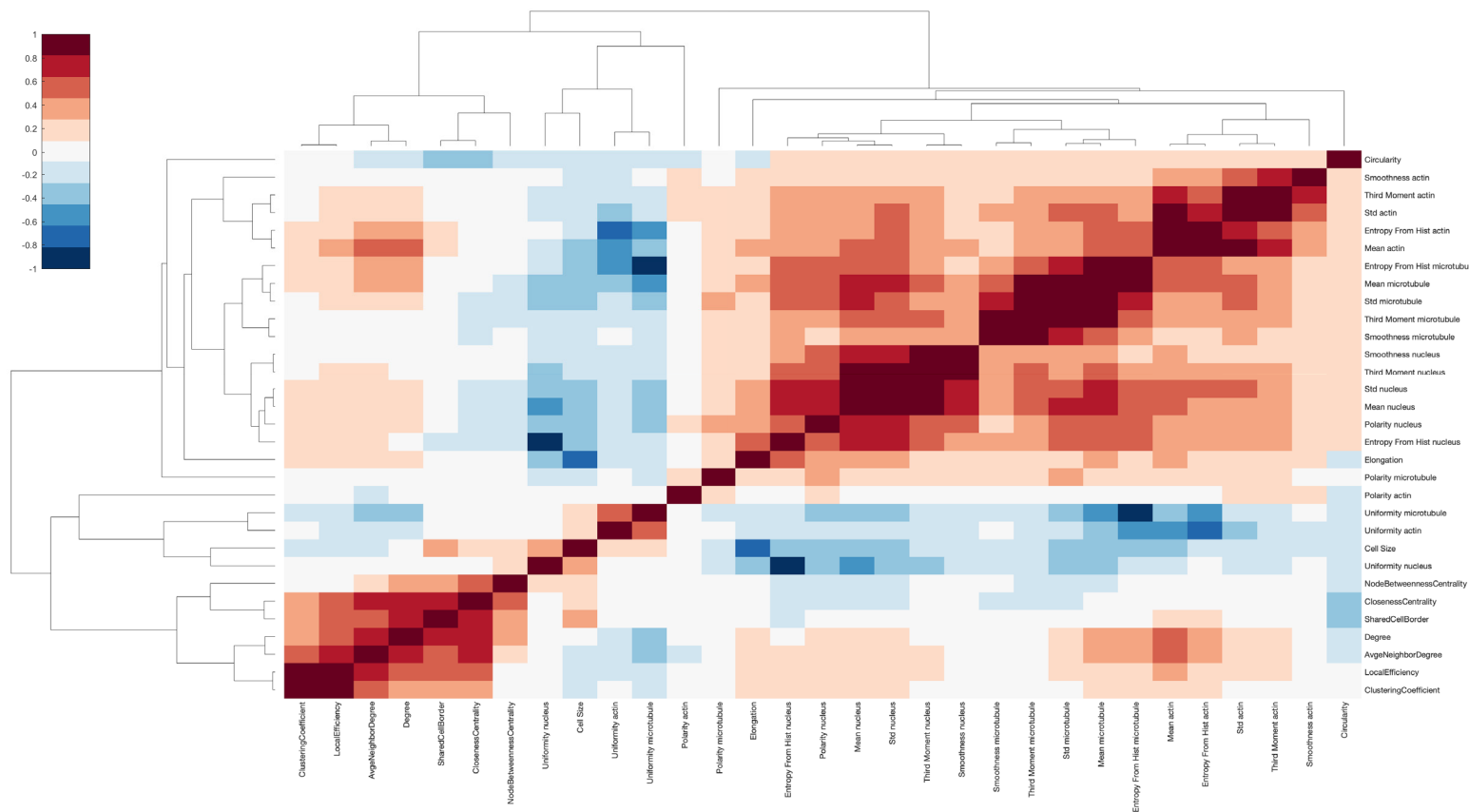

Supplement: S4 Fig — All morphology and local network metrics (S1 and S2 Tables) were combined into a single matrix. The cluster dendrogram was obtained through hierarchical clustering of the covariance matrix using Pearson’s correlation as the similarity metric. (PDF) [file pcbi.1009846.s004.pdf]
