# Supplementary material for: cytoNet: Spatiotemporal network analysis of cell communities
Source: PLoS Comput Biol. 2022 Jun 13;18(6):e1009846. doi: 10.1371/journal.pcbi.1009846 (PMC9191702; doi:10.1371/journal.pcbi.1009846)
Supplement: S1 Table — (PDF) [file pcbi.1009846.s005.pdf]

**Metrics**	**Definition**	**Mathematical Representation**
Cell Size	Cell Spread Area	A_c
Circularity	Shape factor	$\frac{4\pi A_c}{P_c}$<br>Where P_c is perimeter of cell
Elongation	Shape factor	$\frac{P_c}{A_c}$
Polarity*	Distance between center of mass of stain and the centroid of the cell	$\sqrt{(\chi_{C,x} - \Omega_{S,x})^2 + (\chi_{C,x} + \Omega_{S,x})^2}$<br>Where χ_c is centroid of cell and Ω_S is center of mass of stain
Mean*	First moment of grayscale stain intensity distribution	$\sum_{i=0}^{255} \frac{i}{255} \cdot p$<br>Where p is the histogram counts of the image for pixel intensities, with 256 possible bins for a grayscale image
Standard Deviation*	Second moment of grayscale stain intensity distribution	$\sqrt{\sum_{i=0}^{255} \left(\frac{i}{255}\right)^2 \cdot p}$
Third Moment*	Third moment of grayscale stain intensity distribution	$\frac{1}{255^2} \sqrt{\sum_{i=0}^{255} \left(\frac{i}{255}\right)^3 \cdot p}$
Smoothness*	Measure of smoothness of stain	$1 - \frac{1}{1 + \left(\frac{1}{255^2} \sqrt{\sum_{i=0}^{255} \left(\frac{i}{255}\right)^2 \cdot p}\right)}$
Entropy from Histogram*	Measure of randomness of the stain intensity	$-\sum p \cdot log_2(p)$
Uniformity*	Sum of squared elements in the histogram counts of the image for pixel intensities	$\sum p^2$
