# Supplementary material for: cytoNet: Spatiotemporal network analysis of cell communities
Source: PLoS Comput Biol. 2022 Jun 13;18(6):e1009846. doi: 10.1371/journal.pcbi.1009846 (PMC9191702; doi:10.1371/journal.pcbi.1009846)
Supplement: S2 Table — (PDF) [file pcbi.1009846.s006.pdf]

Morphology Metric	Treatment Condition	Cohen's d Effect Size			
		No correction for network metrics		Correction applied for network metrics	
Cell.Size		6hr (uncorrected*)	12hr (uncorrected)	6hr (corrected**)	12hr (corrected)
	BDNF50	0.256	0.217	0.148	0.170
	VEGF50	0.151	0.023	0.093	0.068
Mean.Actin		6hr (uncorrected)	12hr (uncorrected)	6hr (corrected)	12hr (corrected)
	BDNF50	0.381	1.020	0.091	0.873
	BDNF100	0.517	2.522	0.260	1.959
	VEGF50	1.121	1.018	0.348	0.740
	VEGF100	1.267	2.269	0.284	1.808
