# Supplementary material for: cytoNet: Spatiotemporal network analysis of cell communities
Source: PLoS Comput Biol. 2022 Jun 13;18(6):e1009846. doi: 10.1371/journal.pcbi.1009846 (PMC9191702; doi:10.1371/journal.pcbi.1009846)
Supplement: S1 Text — (PDF) [file pcbi.1009846.s011.pdf]

S1 Text: Instructions on using cytoNet

Go to https://www.qutublab.org/how and follow the "cytoNet site" link. An explanation of parameters and input format can also be downloaded there.

1. Select mask files

   a) Select binary mask files by clicking on the 'Browse' button to start a file selection dialog box. Mask file names must start with the prefix "MASK_". Multiple files can be selected by: i) clicking on a file while holding down the control key (command key in MacOS; ii) clicking and dragging; or iii) entering control-a (command-a in MacOS) to select all files in a directory or folder.
   b) For demonstration purposes, cytoNet can provide a mask image if you do not have your own. Check the box next to the sample mask to include it as input.

2. Select edge determination method for spatial graphs

   a) Cell Centroid Distance. Edges between nearby objects are determined by the distance between their centroids. If this method is selected, use the slider bar to specify an adjacency threshold. The adjacency threshold determines the maximum distance between two centroids at which an edge is created in the following way. Let a_1 and a_2 be the area of two objects with centroids c_1 and c_2 respectively. For each object, compute its effective radius: $r_i = \sqrt{a_i/\pi}$. A graph edge is placed between two objects (vertices) whenever the distance between their centroids is within the adjusted sum of their effective radii: $distance(c_1, c_2) \leq S \cdot (r_1 + r_2)$ where S is the user defined adjacency threshold parameter.
   b) Border Overlap. Edges between objects are determined by the sharing of border pixels.
   c) Cell Perimeter Distance. Edges between nearby objects are determined by the shortest distance between pixels of the two objects. If this method is selected, specify the maximum distance in pixels between objects that can be connected by an edge.

3. Select image files (optional)

   a) Select image files by clicking on the 'Browse' button to start a file selection dialog box. Image file names must match mask file names after the "MASK_" prefix is removed. Multiple files can be selected in the same manner as for mask files. Color input images are first converted to grayscale images by cytoNet before being processed as previously described.
   b) If the image files contain sequences of calcium signal images, check the box indicating calcium signaling.
   c) For demonstration purposes, cytoNet can provide a mask image if you do not have your own. Check the box next to the sample image to include it as input.

4. Enter an email address. cytoNet will use this email address to inform you that processing is complete.

5. Click the Submit button.

6. cytoNet will send you an email message indicating that your request has been accepted. This message includes a Request ID that you can use to check on the progress of your request. cytoNet will also send you an email message informing you that processing has ended for your request.

7. When your request has been successfully processed, you may download your results. Note that your results will be available for only a limited amount of time.

Results are formatted as follows. Global metrics are tabulated in a file called 'GlobalMetrics.csv' for all images in the input folder. Local metrics, computed on a per-cell basis are tabulated in a separate file for each image called 'LocalMetrics_filename.csv', where filename is the original file name. Also, basic morphology metrics (size, elongation, circularity and stain intensity) are tabulated in a separate file for each image called SingleCellMetrics_filename.csv, where filename is the original file name. Processed images are also created for each image in the input folder, called 'filename_processed.tif' where the original image is overlaid with cell indices, object outlines (red) and spatial proximity edges (yellow). Cell indices displayed in the processed images are used in the first column of local metrics and single cell metric files.
